# BDH2 triggers ROS-induced cell death and autophagy by promoting Nrf2 ubiquitination in gastric cancer

**DOI:** 10.1186/s13046-020-01620-z

**Published:** 2020-06-30

**Authors:** Jia-Zhou Liu, Yi-Lin Hu, Ying Feng, Yun Jiang, Yi-Bing Guo, Yi-Fei Liu, Xi Chen, Jun-Ling Yang, Yu-yan Chen, Qin-Sheng Mao, Wan-Jiang Xue

**Affiliations:** 1 Department of Gastrointestinal Surgery, Affiliated Hospital of Nantong University, 20 Xisi Street, Nantong, Jiangsu China; 2 Research Center of Clinical Medicine, Affiliated Hospital of Nantong University, 20 Xisi Street, Nantong, China; 3 Department of Pathology, Affiliated Hospital of Nantong University, 20 Xisi Street, Nantong, China

**Keywords:** BDH2, Nrf2, Gastric cancer, ROS, PI3K, Autophagy

## Abstract

**Background:**

3-Hydroxy butyrate dehydrogenase 2 (BDH2) is a short-chain dehydrogenase/reductase family member that plays a key role in the development and pathogenesis of human cancers. However, the role of BDH2 in gastric cancer (GC) remains largely unclear. Our study aimed to ascertain the regulatory mechanisms of BDH2 in GC, which could be used to develop new therapeutic strategies.

**Methods:**

Western blotting, immunohistochemistry, and RT-PCR were used to investigate the expression of BDH2 in GC specimens and cell lines. Its correlation with the clinicopathological characteristics and prognosis of GC patients was analysed. Functional assays, such as CCK-8 and TUNEL assays, transmission electron microscopy, and an in vivo tumour growth assay, were performed to examine the proliferation, apoptosis, and autophagy of GC cells. Related molecular mechanisms were clarified by luciferase reporter, coimmunoprecipitation, and ubiquitination assays.

**Results:**

BDH2 was markedly downregulated in GC tissues and cells, and the low expression of BDH2 was associated with poor survival of GC patients. Functionally, BDH2 overexpression significantly induced apoptosis and autophagy in vitro and in vivo. Mechanistically, BDH2 promoted Keap1 interaction with Nrf2 to increase the ubiquitination level of Nrf2. Ubiquitination/degradation of Nrf2 inhibited the activity of ARE to increase accumulation of reactive oxygen species (ROS), thereby inhibiting the phosphorylation levels of Akt^Ser473^ and mTOR^Ser2448^.

**Conclusions:**

Our study indicates that BDH2 is an important tumour suppressor in GC. BDH2 regulates intracellular ROS levels to mediate the PI3K/Akt/mTOR pathway through Keap1/Nrf2/ARE signalling, thereby inhibiting the growth of GC.

## Background

Gastric cancer (GC) is one of the most common malignant tumours in the world with morbidity and mortality accounting for the fourth and second places among malignant tumours. Each year, more than 800,000 new patients are diagnosed with GC, of which nearly 90% have advanced GC, and few patients are eligible for surgery. Because of the heterogeneity of GC, the efficacy of traditional radiotherapies and chemotherapies is not satisfactory. In recent years, biotherapy and targeted therapy for GC have made great progress, but the prognosis of patients with GC is still not optimistic, and the molecular mechanisms of GC occurrence and development are still unclear [1]. Autophagy is a common physiological process in normal and GC cells. Abnormal levels of autophagy have major effects on the occurrence and progression of GC. Therefore, elucidating the mechanism of autophagy in the development of GC has great clinical significance.

Reactive oxygen species (ROS) are important signalling molecules in cells, which participate in the transmission of information via multiple signalling pathways [2, 3]. Excessive ROS induce tumour cell autophagy and apoptosis by inhibiting PI3K/Akt and other pathways, thereby inhibiting the occurrence and development of tumours [4]. For example, salinomycin promotes autophagy and apoptosis of prostate cancer cells through PI3K/Akt/mTOR and ERK/p38 MAPK pathways by increasing the cellular ROS level [5]. Inhibiting the autophagy level of prostate cancer cells increases their apoptosis level induced by salinomycin, thereby increasing the chemotherapy sensitivity of salinomycin. Ciclopirox olamine increases ROS levels in rectal cancer cells by affecting mitochondrial functions and then induces apoptosis and protective autophagy through the AMPK pathway [6]. Inhibiting this cytoprotective autophagy increases the level of apoptosis in rectal cancer cells induced by ciclopirox olamine. ROS in tumour cells are not only produced by stimulation from the external environment, but are also generated by the cell itself or as a byproduct of other biological reactions [7–9]. Post-translational modifications of proteins, such as ubiquitination and phosphorylation, play an important role in this process. For example, changes in the level of nuclear factor erythroid 2-related factor 2 (Nrf2) SUMOylation affect the intracellular ROS level and thus the autophagy level of cells, which ultimately affect the occurrence and development of hepatocellular carcinoma [10].

BDH2 is a short-chain dehydrogenase/reductase family member originally named as DHRS6 [11]. The human BDH2 gene is located on 4q and encodes 245 amino acids. BDH2 is widely expressed in the cytoplasm of epithelial cells of organs such as the kidney, small intestines, oral cavity, and breast. BDH2 may be a multifunctional gene in mammalian cells. It is a novel cytosolic-type 2-hydroxybutyrate dehydrogenase and has a physiological role in the use of cytosolic ketone bodies that can subsequently enter mitochondria and the tricarboxylic acid cycle [12]. BDH2 also catalyses the synthesis of 2,5-dihydroxybenzoic acid during biosynthesis of enterobactin [13]. In addition, BDH2 may be an independent poor prognosis marker of acute myeloid leukaemia by affecting apoptosis. BDH2 is regulated by long-chain non-coding RNA TP73-AS1, which affects oesophageal squamous cell carcinoma cell proliferation and apoptosis [14]. However, the effect of BDH2 expression on autophagy of GC cells and its possible mechanism have not been reported.

In this study, we analysed the expression level of BDH2 in paired GC tissues by immunohistochemical analysis, and the correlation between BDH2 expression levels and clinicopathological features and prognosis of GC. We found that low expression of BDH2 is an independent molecular marker of a poor prognosis of GC patients. GC cell lines stably expressing BDH2 were constructed, and the effect of the change of the BDH2 expression level on the apoptosis and autophagy of GC cells was observed. On the molecular level, we demonstrated that BDH2 regulates the Keap1-Nrf2 interaction and promotes proteasomal degradation of Nrf2. Increasing the levels of ubiquitination/degradation of Nrf2 led to intracellular ROS accumulation, thereby altering the PI3K/Akt /mTOR pathway to inhibit the growth of GC cells in vivo and in vitro.

## Materials and methods

### Patients and tissue samples

We collected 171 paired tumour and adjacent non-cancerous gastric tissues from January 2010 to December 2010 at the Affiliated Hospital of Nantong University (Jiangsu, China). Detailed clinicopathological information is provided in Table 1. Another cohort of 30 fresh GC cases was collected from the Department of General Surgery for western blot analysis. All patients were definitively diagnosed with GC by pathological histology and had not received adjuvant chemotherapy, radiation therapy, or immunotherapy before surgical excision of tumours. All experiments were conducted with the approval of the Committee for the Ethical Review of Research involving Human Subjects at Nantong University Affiliated Hospital. Written informed consent was obtained from each patient before sample collection.

**Table 1:** Correlation between BDH2 expression in GC tissues and clinicopathological features of GC patients

Clinicopathological parameter	N	BDH2 expression		*p value*
		Low 127	High 44	
**Gender**				0.371
Male	111	80(72.1%)	31(27.9%)	
Female	60	47(78.3%)	13(21.7%)	
**Age (years)**		.	.	0.433
≤60	73	52(71.2%)	21(28.8%)	
> 60	98	75(76.5%)	23(23.5%)	
**Degree of differentiation**				0.177
Well	18	11(61.1%)	7(38.9%)	
Moderate/Poor	153	116(75.8%)	37(24.2%)	
**Tumor diameter (cm)**				0.124
< 5	112	79(70.5%)	33(29.5%)	
≥5	59	48(81.4%)	11(18.6%)	
**Tumor localization**				0.536
Up	19	13(68.4%)	6(31.6%)	
Middle/Down	152	114(75.0%)	38(25.0%)	
**TNM stage**				0.021
I + II	95	64(67.3%)	31(32.7%)	
III	76	63(82.9%)	13(17.1%)	
**Depth of invasion**				0.008
T1 + T2	72	46(63.8%)	26(36.2%)	
T3 + T4	99	81(81.8%)	18(18.2%)	
**Lymph node metastasis**				0.047
Negative	79	53(67.1%)	26(32.9%)	
Positive	92	74(80.4%)	18(19.6%)	

### Cell culture and reagents

Seven GC cell lines (AGS, BGC823, MGC803, MKN45, MKN1, SGC7901, and HGC27), a gastric mucosa cell line (GES-1), and human kidney cell line (HEK293T) were purchased from GeneChem (Shanghai, China). Cells were cultured in RPMI-1640 medium supplemented with 10% FBS at 37 °C in a humidified atmosphere with 5% CO_2_.

DAPI, Baf-A1, and 3-MA were obtained from Solarbio (Beijing, China). NAC was purchased from Beyotime (Shanghai, China), and 740Y-P (also known as 740YPDGFR) was obtained from MCE (Monmouth Junction, NJ, USA).

### Plasmids and cell transfection

FLAG-tagged BDH2, HA-tagged Keap1, and MYC-tagged Nrf2 overexpression plasmid were purchased from GeneChem (Shanghai, China). To construct the BDH2 expression vector, the full-length open reading frame of human BDH2 cDNA was cloned into the eukaryotic expression vector pcDNA3.1 (Invitrogen). Expression vectors were transfected into cells with low expression of BDH2 (SGC7901 and BGC823) using Lipofectamine 3000 (Invitrogen). Stably transfected cells were selected for more than 2 weeks using neomycin (G418; Roche, Indianapolis, IN, USA).

### Cell proliferation, clonogenic assay, cell cycle analysis, and apoptosis assay

These experiments were performed as described previously [15].

### Immunofluorescence staining

Immunofluorescence staining was performed as described in our previous study [15]. The cells were incubated with anti-LC3B (1:1000, Abcam, ab48394) and anti-Nrf2 (1:200, Proteintech, 16,396) antibodies at 4 °C overnight. After washing three times with PBS, the cells were stained with secondary antibodies (ABclonal) at 37 °C for 2 h. Finally, Nuclei were labelled with 0.1 g/mL DAPI for 15 min, except when visualising autophagosomes (LC3-II) using an LSM780 laser-scanning confocal microscope (Carl Zeiss). Other images were captured under a BX41 fluorescence microscope (Olympus, Japan).

### Transmission electron microscopy (TEM)

As described previously [15], transfected cells and xenografted tumour tissues were fixed in glutaraldehyde, and then ultrathin sections were prepared with an EM UC7 ultramicrotome (Leica, Solms, Germany). The sections were then imaged by a transmission electron microscope (Hitachi Scientific Instruments, Mountain View, CA, USA) to detect autophagic vacuoles.

### Immunohistochemistry (IHC)

The IHC protocol was performed as described previously [15]. Briefly, tumour sections of patients and nude mouse xenografts were analysed by IHC using the EnVision™ System (Dako, Carpinteria, CA). Primary antibodies used for IHC were anti-BDH2 (1:100, Proteintech, 27,207), anti-Ki67 (1:100, Abcam, ab15580), anti-cleaved caspase-3 (1:200, CST, #9661), anti-LC3B (1:200, Abcam, ab48394), anti-Nrf2 (1:50, Proteintech, 16,396), anti-p-Akt (1:100, Proteintech, 66,444), and anti-p-mTOR (1:100, Abcam, ab109268).

### In vivo tumourigenesis assays

Male BALB/c nude mice were randomly divided into two groups (*n* = 6/group). Two groups of mice were subcutaneously injected with 1 × 10^7^ cells transfected with an empty vector or BDH2 expression vector into the right side of the neck. After tumours were visible, the tumour size was measured every 3 days until 30 days. The formula volume = (length×width^^2^)/2 cm^2^ was used to calculate tumour volumes. The mice were sacrificed after 30 days, and the tumour weight was measured. The xenografted tumours were fixed for histological analysis. A TUNEL assay was performed using an In Situ Cell Death Detection Kit (Roche; Mannheim, Germany). The Animal Care Committee of Nantong University reviewed and approved all animal experiments.

### Reactive oxygen species (ROS) assay

Measurement of intracellular ROS was performed using 2,7-dichlorofluorescin-diacetate (DCFH-DA) (Yeasen, Shanghai, China). Cells were incubated with 10 μM DCFH-DA diluted in RPMI-1640 medium for 30 min at 37 °C in the dark. The cells were then immediately observed under the fluorescence microscope. Fluorescence intensity was quantified using ImageJ software. For flow cytometry, cells were collected for analysis. For tissue ROS measurement, frozen tumour sections were incubated with DCFH-DA (Item No: E004, Jiancheng Bioengineering, Nanjing, China) at 37 °C for 30 min. The ROS level in the tissue was measured under the BX41 fluorescence microscope.

### Autophagic flux analysis

As described previously [15], cells were transfected with GFP-mRFP-LC3 (Shanghai, China) for 24 h and then treated for 48 h with or without 10 nM Baf-A1. Images were obtained under a confocal microscope (Carl Zeiss).

### Quantitative real-time PCR (qRT-PCR)

Total RNA in GC cells was extracted with Trizol reagent (Invitrogen). qRT-PCR was carried out as described previously [16]. Primer sequences are listed in Table S1.

### Transient transfection and luciferase assay

A pGL3-ARE-Luc reporter was obtained from Genechem, which is a ready-to-transduce ARE responsive lentiviral firefly luciferase reporter to monitor the transcriptional activity of Nrf2. Cells were cultured in 6-well plates and then transfected with the pGL3-ARE-Luc reporter plasmid and pRL-TK plasmid (Promega) using Lipofectamine 3000. A Luciferase Reporter Gene Assay Kit (Beyotime Institute of Biotechnology) was used to determine ARE-driven promoter activity. Luciferase activity is expressed as the ratio to that in control cells.

### Coimmunoprecipitation (co-IP)

Total cell lysates were incubated with 1 μg primary antibody or negative control rabbit IgG at 4 °C overnight. Then, 20 μl protein A + G agarose (Bioworld Technology, St. Louis Park, MN, USA) was added, followed by further incubation for 2 h at 4 °C. Protein–antibody complexes were rinsed four times with PBS, and the beads were collected by centrifugation. Proteins were detected by SDS-polyacrylamide gel electrophoresis and western blotting.

### Western blotting

Total protein separation and western blotting were performed as described previously [16]. Western blot analysis was performed using anti-cleaved PARP (#5625), anti-cleaved caspase-3 (#9661), anti-p-AMPK (Thr172) (#50081), anti-p-ERK1/2 (#4370), and anti-HA-Tag (#3724) antibodies purchased from Cell Signaling Technology (Danvers, MA, USA). Anti-LC3 (14600–1-AP), anti-P62/SQSTM1 (18420–1-AP), anti-phospho-Akt (Ser-473) (66444–1-Ig), anti-Akt (10176–2-AP), anti-mTOR (20657–1-AP), anti-P38 MAPK (14064–1-AP), anti-JNK (51151–1-AP), anti-ERK1/2 (16443–1-AP), anti-AMPK (10929–2-AP), anti-Nrf2 (16396–1-AP), anti-Keap1 (10503–2-AP), anti-FLAG-tag (66008–2-AP), anti-MYC-tag (60003–2-AP), and anti-β-actin (66009–1-Ig) antibodies were obtained from Proteintech Group Co., Ltd. (Wuhan, China). An anti-phospho-mTOR (Ser-2448) (ab109268) antibody was purchased from Abcam (Cambridge, MA, USA). Anti-p-p38 (sc-7973), anti-p-JNK (sc-6254), and anti-Ub (sc-8017) antibodies were obtained from Santa Cruz Biotechnology., Inc. (Santa Cruz, CA, USA).

### Ubiquitination assay

For the polyubiquitinated Nrf2 assay, whole cell lysates prepared with RIPA buffer containing a proteinase inhibitor were subjected to IP of endogenous Nrf2 protein. The levels of Nrf2 ubiquitination were detected by immunoblotting with an anti-Ub antibody.

#### Assessment of intracellular Iron levels

Iron levels were measured by Iron Colorimetric Assay Kit (ABIN411680, Biovision) according to the manufacturer’s instructions [13].

### Statistical analysis

Data are presented as means ± standard deviation (SD). All experiments were performed at least three times. Statistical analysis was performed with the SPSS software version 22 and GraphPad Prism 7.0 software. The survival rates patients with GC were calculated by Kaplan-Meier and log-rank analyses, and prognostic factors were evaluated using the Cox regression model. Student’s t-test was used for statistical comparisons between experimental groups. *p* < 0.05 was considered as statistically significant.

## Results

### BDH2 is downregulated in GC tissues

**Fig. 1:**
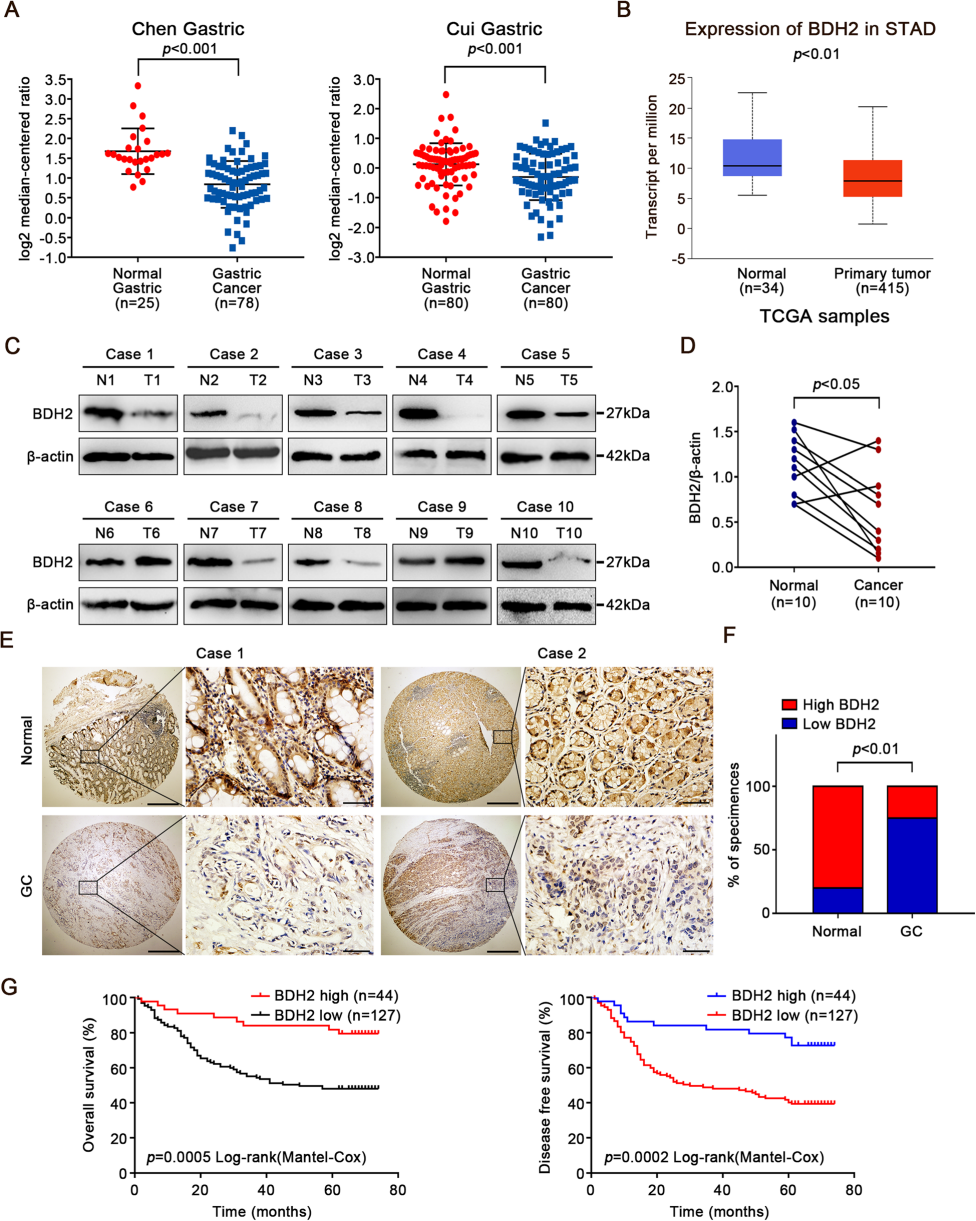
Expression and prognostic value of BDH2 in GC. **a**, **b** Studies in the Oncomine and UALCAN database showed lower mRNA expression of BDH2 in GC tissues. **c** Expression levels of BDH2 protein were detected in 10 matched GC samples by western blotting (N, normal tumour-adjacent tissue; T, tumour tissue). **d** Quantification of BDH2 expression in GC tissues and adjacent normal tissues. **e** Expression of BDH2 was analysed by a TMA including 171 GC cases. Typical images are shown. Scale bars, 50 μm. **f** IHC quantification scores of BDH2 expression levels. **g** Kaplan–Meier survival curves illustrating the overall survival and disease-free survival of patients with GC associated with BDH2 expression (*p* < 0.001)

To investigate the role of BDH2 in GC, we first analysed the BDH2 mRNA level in the Oncomine database (https://www.oncomine.org) and found that its expression was significantly lower in GC tissues compared with corresponding normal tissues (Fig. 1a). We also observed a similar down-regulation of BDH2 expression in GC in the UALCAN dataset (http://ualcan.path.uab.edu/index.html) (Fig. 1b). We next analysed BDH2 protein expression in matched gastric normal (N) and cancerous (T) tissues by western blotting. As shown in Fig. 1c, d, lower BDH2 expression was observed in eight of 10 GC cases compared with matched adjacent normal tissues (*p* < 0.05). Immunohistochemical analysis was subsequently performed on a TMA of 171 GC and matched adjacent normal samples. Low protein expression of BDH2 was observed in 74.3% (127/171) of GC tissues compared with matched adjacent non-cancerous tissue samples (*p* < 0.01; Fig. 1e and f). These results suggest that BDH2 expression is downregulated in GC.

We then analysed the BDH2 expression status together with various clinicopathological features of the 171 GC patients (Table 1). The results showed that low expression of BDH2 was positively associated with the TNM stage, depth of invasion, and lymph node metastasis, but not with other clinicopathological features including gender, age, differentiation, tumour localization, or tumour size. Moreover, multiple logistic regression analysis showed that low expression of BDH2 was significantly correlated with depth of invasion (*p* < 0.05, odds ratio [OR] = 0.461, 95% confidence interval [CI]: 0.214–0.993).

In addition, Kaplan-Meier analysis showed that GC patients with low BDH2 expression had worse OS and DFS rates than those with high BDH2 expression (Fig. 1g). Moreover, Cox multivariate analysis showed that low expression of BDH2 was an independent predictor of OS and DFS (Table 2).

**Table 2:** Univariate and multivariable analysis of OS and DFS of patients with GC

Variable	OS			DFS		
	Univariate analysis *P* > |z|	Multivariable analysis		Univariate analysis *P* > |z|	Multivariable analysis	
		*P* > |z|	HR (95% CI)		*P* > |z|	HR (95% CI)
**Gender**						
Male (*n* = 111) vs. female (*n* = 60)	0.290			0.264		
**Age (years)**						
≤ 60 (*n* = 73) vs. > 60 (*n* = 98)	0.787			0.805		
**Differentiation**						
Well (*n* = 18) vs. Moderate/Poor (*n* = 153)	0.075			0.073		
**Tumor diameter (cm)**						
< 5 (*n* = 112) vs. ≥5 (59)	0.015			0.019		
**Tumor localization**						
Up (*n* = 19) vs. Middle/Down (152)	0.425			0.429		
**Depth of invasion**						
T1 + T2 (*n* = 72) vs. T3 + T4 (*n* = 99)	< 0.001	0.003	2.431(1.346-4.390)	< 0.001	0.004	2.367(1.311-4.274)
**Lymph node metastasis**						
Negative (*n* = 79) vs. Positive (*n* = 92)	< 0.001	0.001	2.642(1.513-4.615)	< 0.001	0.001	2.653(1.518-4.637)
**BDH2 expression**						
Low (*n* = 127) vs. High (*n* = 44)	0.001	0.011	0.404(0.200-0.815)	0.001	0.017	0.426(0.211-0.861)

### Ectopic expression of BDH2 inhibits GC via inhibiting cell proliferation and inducing cell cycle arrest and apoptosis

**Fig. 2:**
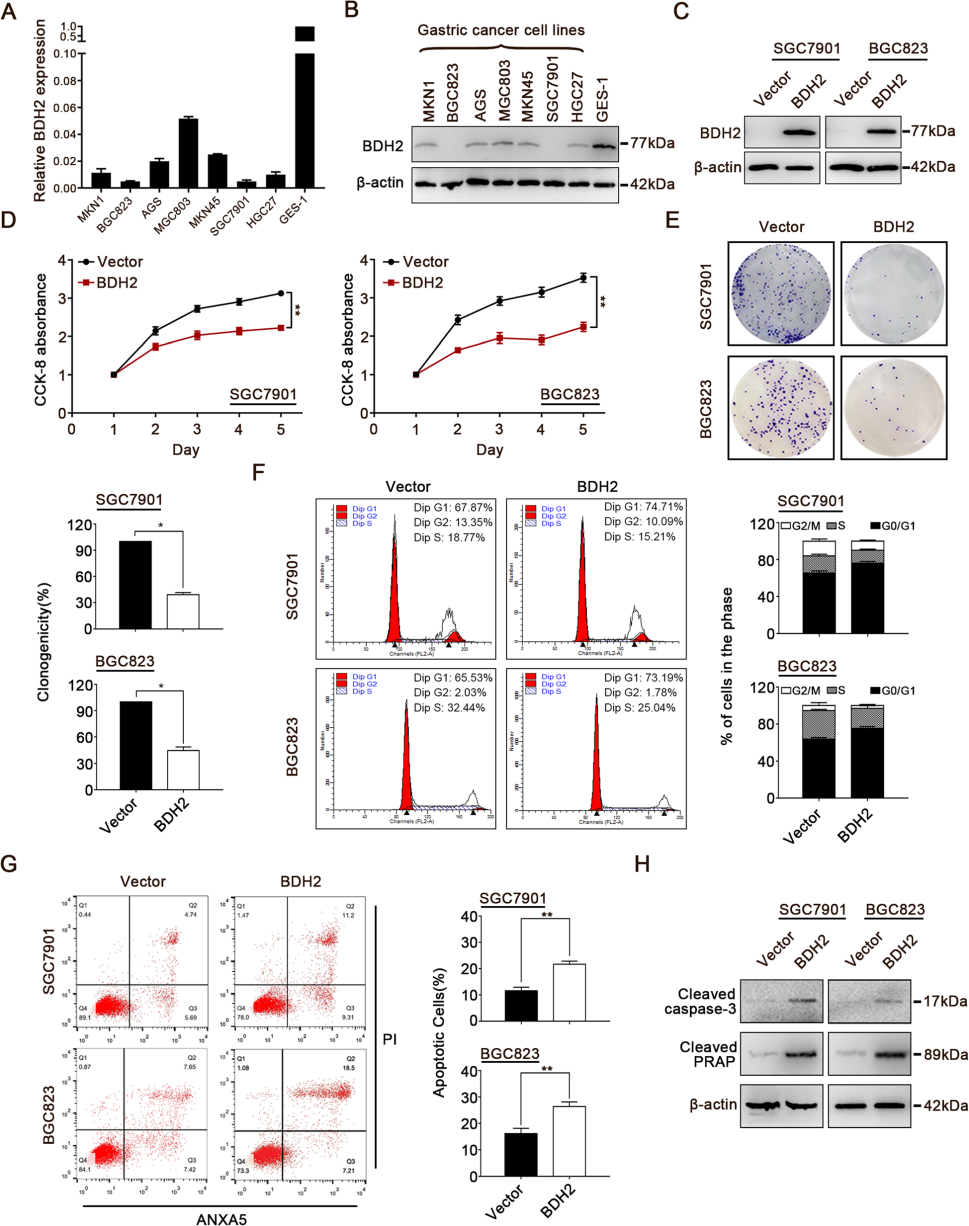
Effect of BDH2 on cell growth. **a**, **b** BDH2 was silenced or downregulated in GC cell lines, but had steady expression in normal gastric tissue as determined by qRT-PCR and western blot analyses. **c** SGC7901 and BGC823 cells were transfected with an empty vector or BDH2 expression vector to establish stably BDH2-overexpressing cell lines. Exogenous expression of BDH2 was detected by western blotting. **d** CCK-8 assays showed that BDH2 significantly inhibited cell viability. **e** Colony formation abilities of SGC7901 and BGC823 cells were markedly hampered by BDH2 overexpression. **f** Flow cytometry was performed to detect the effects of BDH2 on the cell cycle. **g** Flow cytometry was used to evaluate the effect of BDH2 on apoptosis. Apoptotic cells were detected by ANXA5 and PI staining. **h** Expression levels of apoptosis markers in BDH2-expressing cells were confirmed by western blot analysis. Results are presented as means ± S.D. (*n* = 3); *p < 0.05, **p < 0.01

Real-time PCR and western blot analyses were performed to investigate the mRNA and protein levels of BDH2 in seven GC cell lines and GES-1 cells. The BDH2 mRNA and protein levels in GC cell lines were significantly lower than those in GES-1 cells (Fig. 2a and b). To elucidate the biological functions of BDH2 in GC, SGC7901 and BGC823 cells with the lowest endogenous levels of BDH2 were stably transfected with the BDH2 expression vector. Re-expression of BDH2 protein in these cells was confirmed by western blotting (Fig. 2c). Overexpression of BDH2 significantly inhibited cell proliferation as evidenced by CCK-8 and clonogenic assays of SGC7901 and BGC823 cells (Fig. 2d and e).

To examine the molecular mechanism of BDH2-mediated inhibition of cell growth, we determined the effects of BDH2 on cell cycle progression and apoptosis by flow cytometry. The results showed that BDH2 overexpression resulted in significant cell cycle arrest at G0/G1 phase and induced apoptosis (Fig. 2f and g). To confirm the effects of BDH2 on apoptosis, apoptosis markers were analysed by western blotting. The results indicated that expression of cleaved caspases-3 and cleaved PARP was significantly increased in BDH2-overexpressing cells (Fig. 2h). Taken together, these results indicated that overexpression of BDH2 significantly inhibited the growth of GC cells.

### BDH2 inhibits tumour growth in vivo

**Fig. 3:**
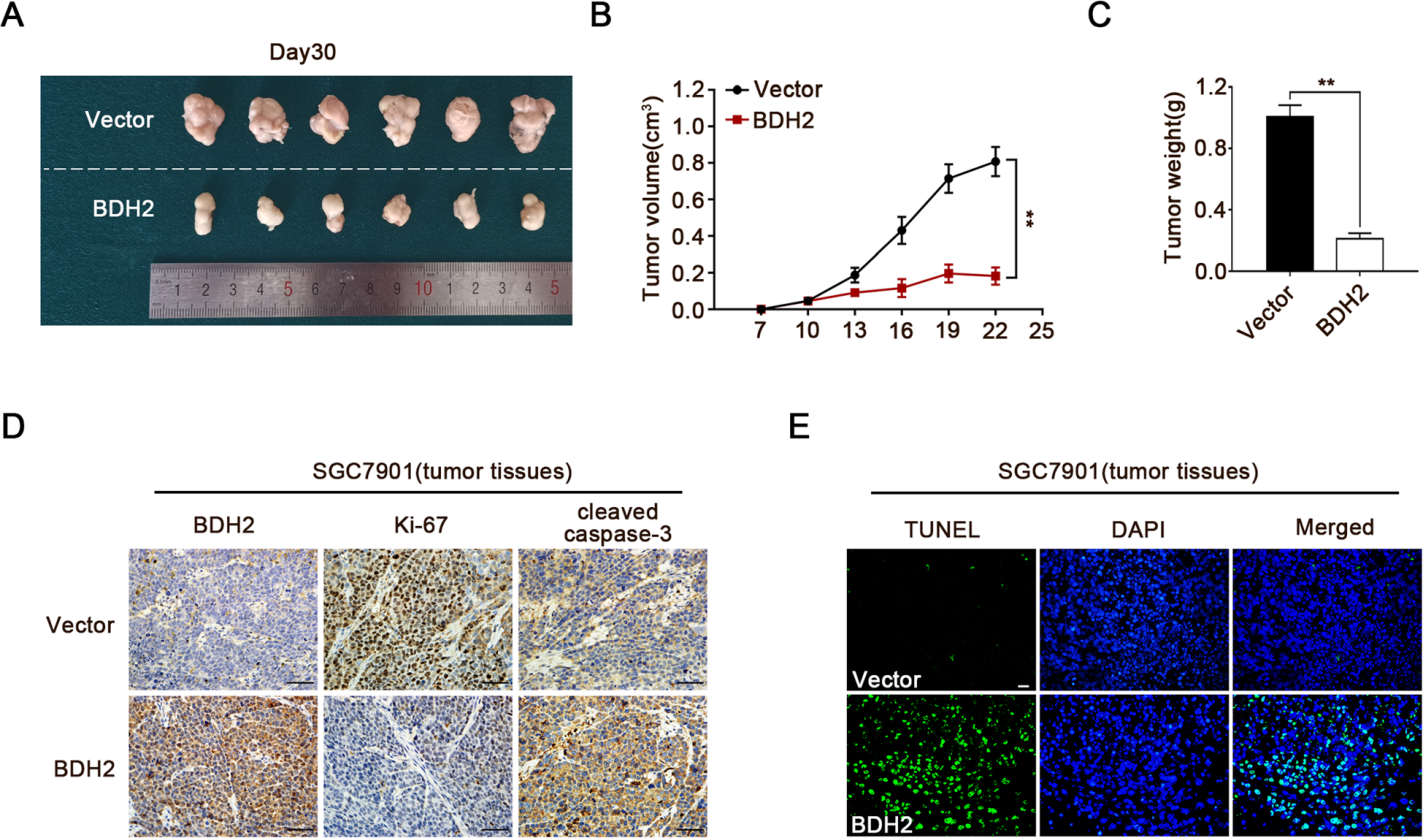
Effect of BDH2 on tumourigenicity in vivo. **a** Representative images of tumours formed by SGC7901 BDH2-overexpressing or vector control cells on day 30. **b** Tumour growth curves. **c** Tumour weight is the mean of three independent experiments. **d** Levels of BDH2, Ki-67, and cleaved caspase-3 were detected by IHC. Scale bars, 50 μm. **e** Tumour sections were stained with TUNEL to count apoptotic cells. Scale bars, 20 μm

To investigate the effect of BDH2 on GC growth in vivo, a nude mouse xenograft model was established by subcutaneous injection of BDH2- or empty vector-transfected SGC7901 cells. As shown in Fig. 3a-c, the volume and net weight of tumours expressing BDH2 were significantly smaller compared with the empty vector group. Moreover, immunohistochemical analysis of the xenografts showed that BDH2 overexpression reduced the expression of Ki-67 and enhanced the expression of cleaved caspase-3 (Fig. 3d). TUNEL analysis also indicated that the apoptosis rate was increased in the BDH2-expressing group (Fig. 3e). These results demonstrated that BDH2 overexpression inhibits GC growth in vivo.

### BDH2 induces autophagy in GC cells

**Fig. 4:**
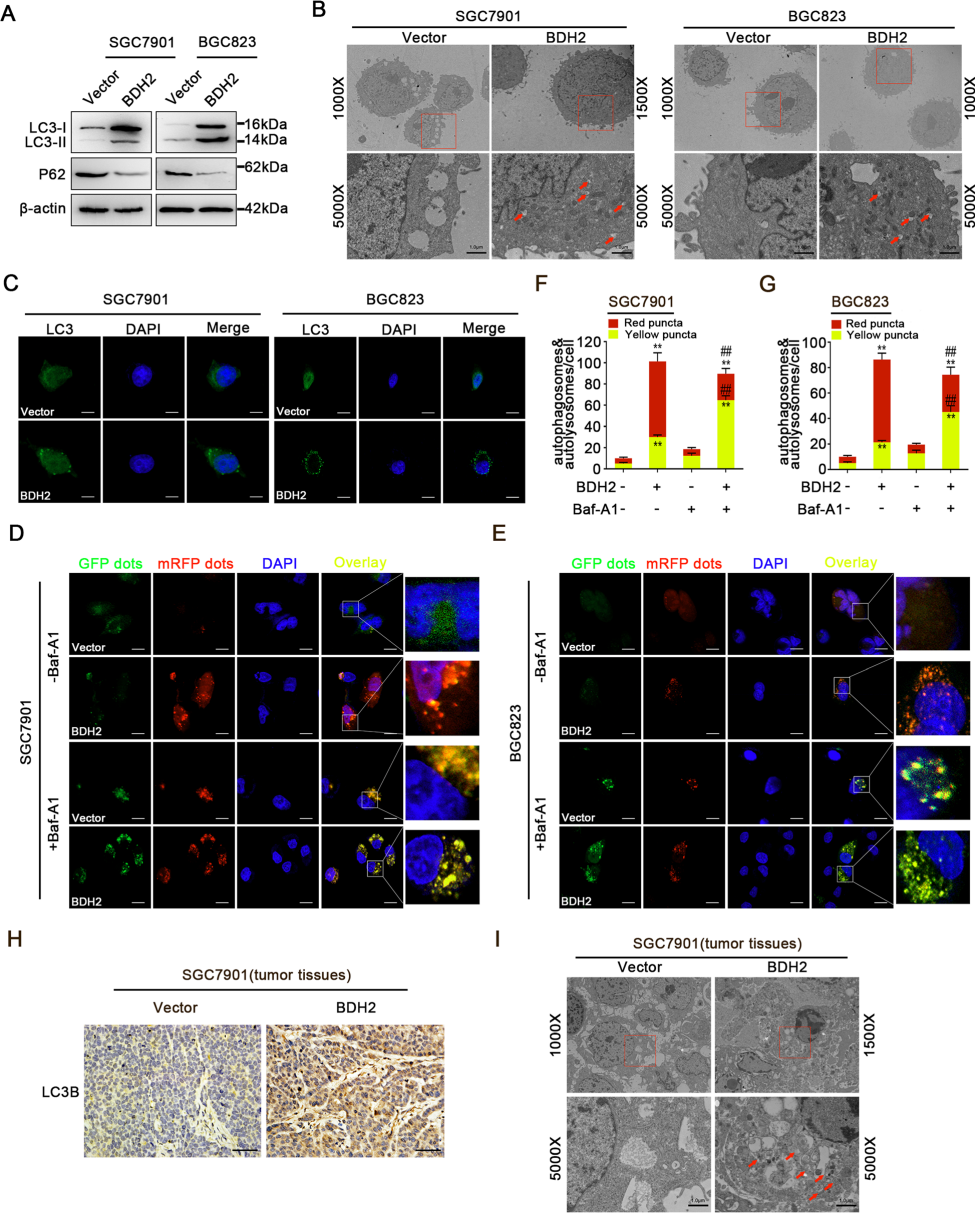
BDH2 induces autophagy in GC cells. **a** Expression levels of LC3-II and p62 in BDH2-overexpressing cells were detected by western blotting. **b** TEM images of endogenic autophagic microstructures in BDH2-overexpressing and vector control cells. Red arrows indicate autophagosomes or autolysosomes. Scale bars, 1 μm. **c** Formation of endogenous LC3 puncta was detected by fluorescence microscopy. Scale bars, 20 μm. **d**, **e** BDH2-overexpressing and vector control cells were infected with an mRFP-GFP-LC3 lentivirus. **f**, **g** Numbers of LC3 puncta (yellow puncta for autophagosomes, red puncta for autolysosomes) were calculated. **h** Expression of LC3B in tumours was detected by IHC. Scale bars, 50 μm. **i** Xenografted tumours were subjected to TEM. Results are presented as means ± S.D. (*n* = 3); *p < 0.05, **p < 0.01 compared with vector; ^##^*p* < 0.01 compared with BDH2 transfected cells treated with Baf-A1

Autophagy is a type of programmed cell death in addition to apoptosis. To examine whether the cytotoxic effect of BDH2 resulted from autophagy activation, we detected the expression levels of two autophagy-related proteins by western blotting. As shown in Fig. 4a, BDH2 overexpression potently increased the protein level of LC3-II, but downregulated the protein expression level of p62. In addition, TEM showed that formation of autophagic vacuoles was frequent in BDH2-overexpressing cells (Fig. 4b). Furthermore, endogenous LC3 puncta were remarkably increased in BDH2-overexpressing GC cells (Fig. 4c). After GFP-mRFP-LC3 lentivirus transfection, the numbers of red puncta (autophagolysosomes) were increased in BDH2-overexpressing SGC7901 and BGC823 cells, indicating an increase in autophagic flux. When bafilomycin A1 (Baf-A1) pretreatment was performed to block autophagic flux, more yellow dots (autophagosomes) were observed (Fig. 4d-g). Furthermore, we investigated the effect of BDH2 overexpression on GC autophagy in vivo. IHC staining indicated that the expression level of LC3B was upregulated in xenografts of BDH2-overexpressing cells (Fig. 4h). Moreover, TEM results demonstrated that BDH2 overexpression significantly increased autophagic double-membrane compartments containing lamellar structures (Fig. 4i). These data indicated that BDH2 overexpression induced autophagy in GC cells.

### BDH2 induces cell death by promoting autophagosome formation in GC cells

There is increasing evidence of a complex link between autophagy and apoptosis. To analyse whether there was an intrinsic link between BDH2-induced GC apoptosis and autophagy, we pretreated SGC7901 and BGC823 cells with 3-methyladenine (3-MA; an autophagy inhibitor) that prevents the formation of autophagosomes. The results showed that 3-MA treatment not only inhibited BDH2 overexpression-induced autophagy, but also decreased BDH2-induced apoptosis caused by active caspase-3 and cleaved PARP (Fig. S1A). The viability of BDH2-overexpressing cells was decreased significantly after treatment with 3-MA (Fig. S1B). In addition, 3-MA treatment inhibited overexpressed BDH2-induced GC cell apoptosis (Fig. S1C). These data revealed that autophagy induced by BDH2 had a pro-death function in GC cells.

### BDH2 induces apoptosis and autophagy via ROS in GC cells

**Fig. 5:**
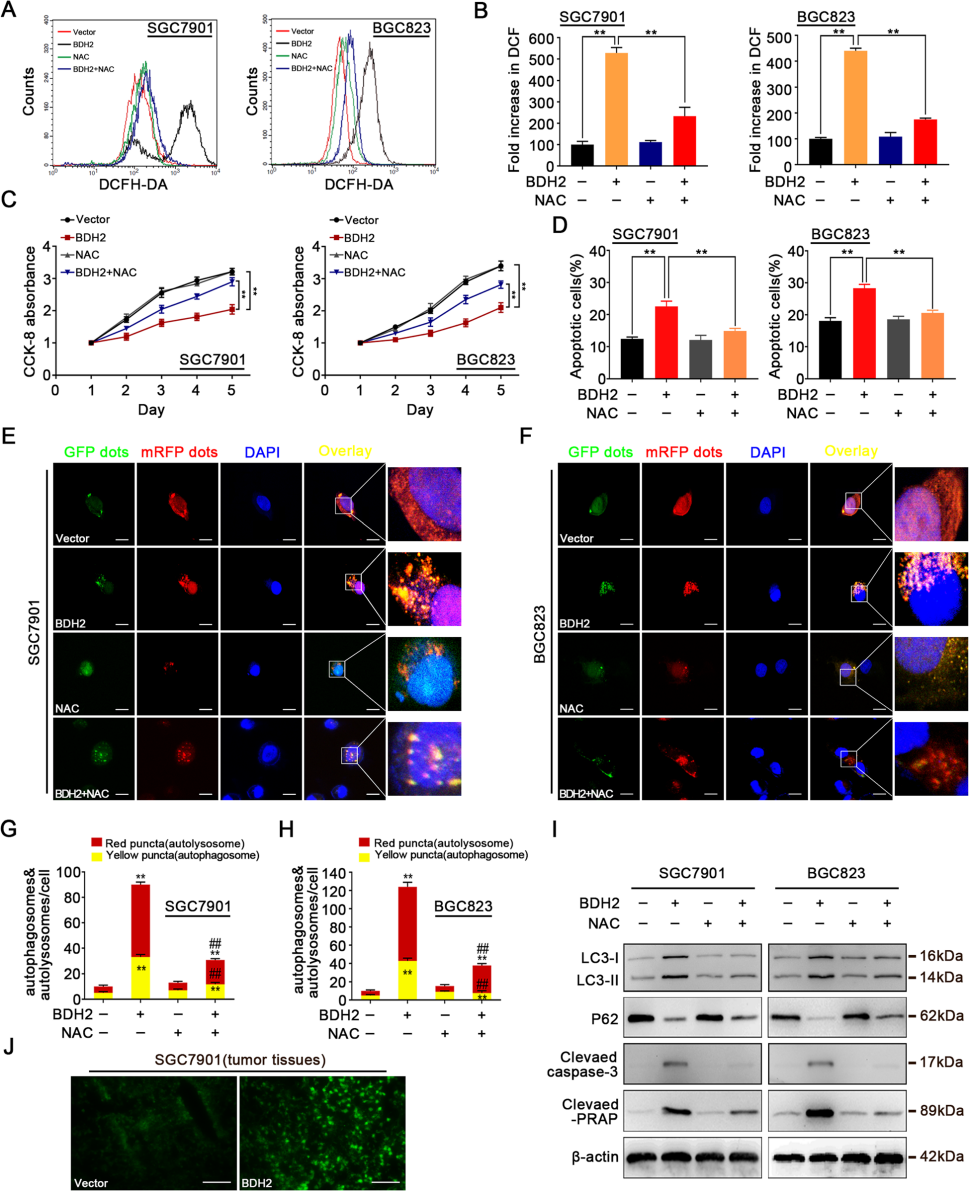
ROS induced by BDH2 overexpression promotes apoptosis and autophagy in GC cells. **a–i** BDH2-overexpressing cells with or without NAC treatment. **a** BDH2-overexpressing and vector control cells were stained with DCFH-DA to measure intracellular ROS production by flow cytometry. **b** Quantitative analysis of ROS generation. **c** Cell viability was measured by CCK-8 assays. **d** Quantitative analysis of the percentage of apoptotic cells. **e–h** Detection of autophagic flux by the GFP-mRFP-LC3 lentivirus in BDH2-overexpressing cells. Scale bars, 20 μm. **i** Protein expression levels of LC3-II, p62, cleaved caspase-3, and cleaved-PARP were detected by western blotting. **j** Frozen tissue sections were stained with the DCFH-DA probe and tissue ROS levels were observed by fluorescence microscopy. Scale bars, 20 μm. Results are presented as means ± S.D. (*n =* 3); *p < 0.05, **p < 0.01 compared with vector; ^##^*p* < 0.01 compared with BDH2 transfected cells treated with NAC

Previous studies have demonstrated that ROS are responsible for triggering cell death and autophagy in tumours [17–19]. Therefore, we analysed the effects of BDH2 overexpression on ROS levels in GC cells. Flow cytometry indicated that BDH2 overexpression increased ROS levels in GC cells (Fig. 5a and b). Fluorescence microscopy also showed that BDH2 overexpression increased the DCF fluorescence intensity in GC cells (Fig. S2). To examine whether there was an intrinsic link between ROS accumulation caused by BDH2 overexpression and the changes in biological behaviours of GC cells, we pretreated GC cells with the ROS inhibitor N-acetylcysteine (NAC). The pretreatment with NAC inhibited apoptosis (Fig. 5c and d) and autophagy (Fig. 5e-h) in BDH2-overexpressing cells. Moreover, western blot assays showed that pretreatment with NAC reversed the change in expression levels of cleaved caspase-3, cleaved PARP, LC3-II, and p62 in BDH2-overexpressing cells (Fig. 5i). Interestingly, we found that tissue samples derived from the BDH2-treated group showed stronger DCF fluorescence (Fig. 5j). Collectively, these findings revealed that BDH2 induces cellular ROS accumulation, thereby promoting apoptosis and autophagic cell death.

### BDH2 increases the intracellular ROS level in GC cells by enhanced ubiquitination and degradation of Nrf2

**Fig. 6:**
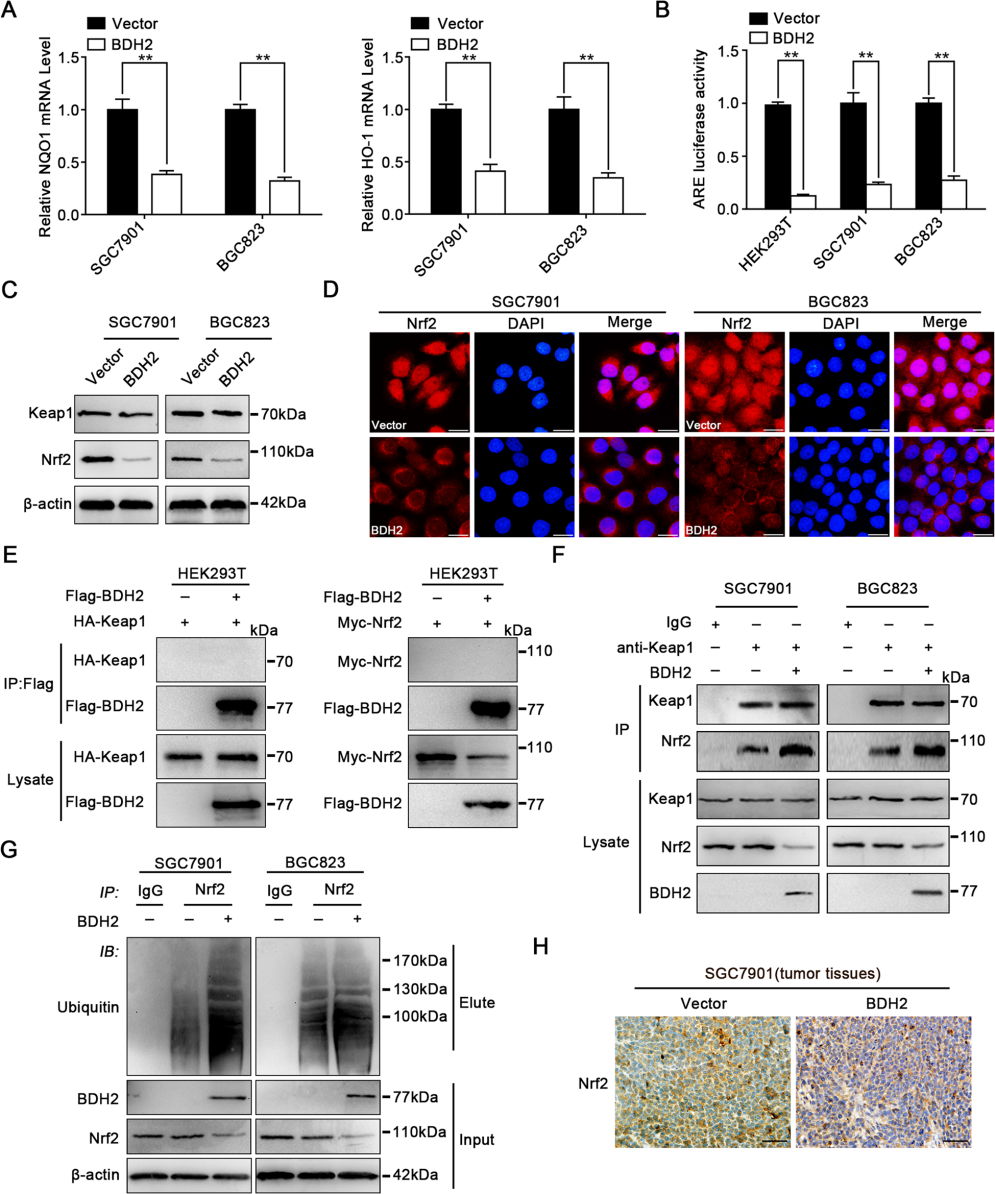
BDH2 suppresses the Keap1/Nrf2/ARE signalling pathway. **a** mRNA expression of ARE-dependent genes was determined by qRT-PCR in the indicated cells. **b** ARE luciferase activity in the indicated cells was determined by a fluorescence microplate reader. **c** Protein expression levels of Keap1 and Nrf2 were determined by western blotting. **d** Fluorescence microscopy showed that accumulation of Nrf2 was inhibited in the nucleus. Scale bars, 20 μm. **e** Co-IP assay with anti-Flag in HEK293T cells expressing HA-tagged Keap1 or Myc-tagged Nrf2, followed by western blotting with antibodies against the HA-tagged Keap1 or Myc-tagged Nrf2. **f** Effect of BDH2 overexpression on binding of Keap1 and Nrf2 was determined by coimmunoprecipitation. Immunoprecipitation with an anti-Keap1 antibody was performed, followed by immunoblotting for Nrf2 and Keap1. **g** BDH2 decreased the Nrf2 level by promoting its ubiquitin-mediated degradation. **h** The level of Nrf2 was examined by IHC. Scale bars, 50 μm. Results are presented as means ± S.D. (*n =* 3); *p < 0.05, **p < 0.01

It has been reported that the kelch-like ECH-associated protein 1 (Keap1)-Nrf2-antioxidant response elements (ARE) signal transduction pathway maintains intracellular redox homeostasis by regulating ROS levels [20]. To investigate the underlying mechanism of ROS accumulation caused by BDH2, we examined the effect of BDH2 on expression of Nrf2 target genes heme oxygenase 1 (HO-1) and NAD(P)H-quinone oxidoreductase 1 (NQO1). qRT-PCR analyses showed that the transcription levels of HO-1 and NQO1 were markedly downregulated in BDH2-overexpressing cells (Fig. 6a). Furthermore, a luciferase reporter plasmid containing an ARE sequence was transfected into HEK293T, SGC7901, and BGC823 cells, and luciferase assays indicated that BDH2 overexpression reduced the activity of the ARE in the cells (Fig. 6b). To reveal the cause of ARE promoter activation, we analysed the expression levels of Keap1 and Nrf2 in BDH2-overexpressing cells by qRT-PCR and western blot. BDH2 did not change the mRNA level of Nrf2 or Keap1 (Fig. S3). However, BDH2 significantly decreased the protein level of Nrf2. Conversely, the protein level of Keap1 was not affected (Fig. 6c). Moreover, immunofluorescence revealed that the level of Nrf2 was significantly decreased in BDH2-overexpressing cells (Fig. 6d). Collectively, these results suggested that BDH2 overexpression downregulates Nrf2 expression through its protein stability rather than transcription.

To gain insight into the molecular mechanistic basis of BDH2 regulating the Keap1-Nrf2 pathway, we overexpressed BDH2 plasmid with Flag-tag, HA-tagged Keap1 and Myc-tagged Nrf2 in HEK293T cell line. After immunoprecipitation using antibody against Flag-tag, HA-tagged Keap1 and Myc-tagged Nrf2 were not detected with anti-HA and anti-Myc antibody, indicating BDH2 failed to interact with Keap1 or Nrf2 (Fig. 6e). Interestingly, Co-IP showed that overexpressing BDH2 increased the interaction of endogenous Nrf2 with Keap1 (Fig. 6f). Previous research has shown that Keap1 mediates proteasomal degradation of Nrf2 by recruiting E3 ubiquitin ligase Cullin 3. To examine whether BDH2 decreased the level of Nrf2 by affecting ubiquitination/degradation of Nrf2, the ubiquitination level of Nrf2 was investigated in BDH2-overexpressing cells. As a result, we found that BDH2 overexpression promoted the ubiquitination of Nrf2 (Fig. 6g). A similar tendency was consistently observed by IHC staining of Nrf2. Nrf2-positive cells were also decreased in BDH2-expressing xenografts (Fig. 6h). These results revealed that BDH2 reduced the Nrf2 protein level by inhibiting its dissociation from E3 ubiquitin ligase Keap1, thereby reducing activity of the ARE promoter and correspondingly downregulating the transcription levels of HO-1 and NQO1.

### ROS/PI3K/Akt/mTOR axis contributes to BDH2-induced apoptosis and autophagy initiation

**Fig. 7:**
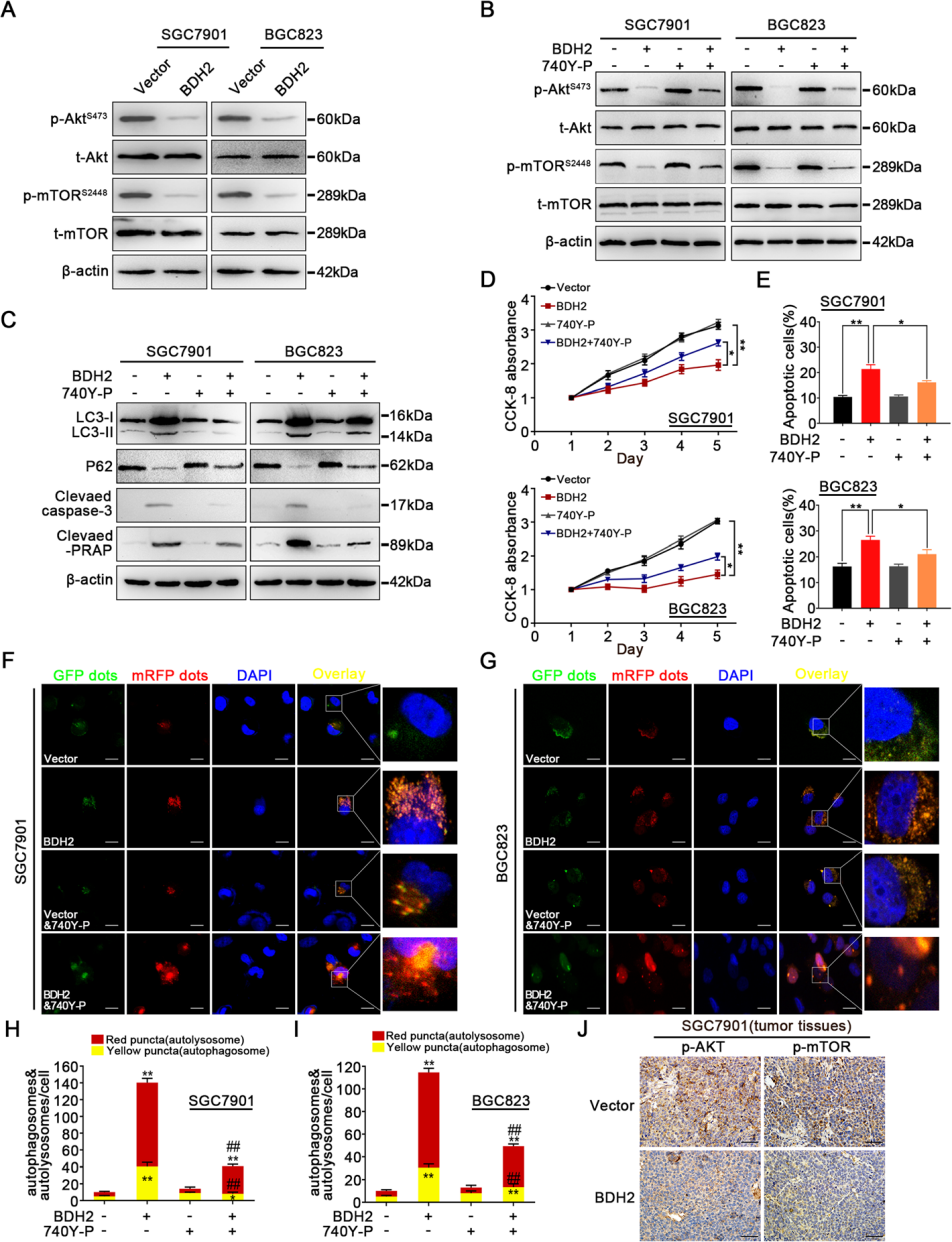
ROS/PI3K/Akt/mTOR axis plays a key role in BDH2-induced apoptosis and autophagy. **a** Levels of p-Akt^Ser473^ and p-mTOR^Ser2448^ were examined by western blotting in BDH2-overexpressing and vector control cells. **b**, **c** Protein expression levels of p-Akt^Ser473^, p-mTOR^Ser2448^, LC3-II, p62, cleaved caspase 3, and PARP with or without 740Y-P treatment were measured by western blotting. **d** Viabilities of SGC7901 and BGC823 cells were detected by CCK-8 assays. **e** Percentages of apoptotic cells were examined by ANXA5 and PI staining. **f–i** Detection of autophagic flux by the GFP-mRFP-LC3 lentivirus in BDH2-overexpressing cells. Scale bars, 20 μm. **j** p-Akt and p-mTOR expression in xenografted tumours was examined by IHC staining. Scale bars, 50 μm. Results are presented as means ± S.D. (*n* = 3); *p < 0.05, **p < 0.01 compared with vector; ^##^*p* < 0.01 compared with BDH2 transfected cells treated with 740Y-P

To further explore the underlying mechanism of BDH2-induced apoptosis and autophagy, we examined the effect of BDH2 on the expression of classic autophagy-related pathways such as AMPK, MAPK/Erk1/2, and PI3K/Akt/mTOR signalling [21, 22]. As shown in Fig. 7a and Fig. S4A, compared with the empty vector group, no significant changes in the expression of AMPK or MAPK/Erk1/2 were found in BDH2-overexpressing cells. However, the expression levels of p-Akt^Ser473^ and p-mTOR^Ser2448^ were significantly lower than in the vector group (Fig. 7a; Fig. S4A). Pretreatment with 740Y-P (PI3K agonist) inhibited downregulation of p-Akt^Ser473^ and p-mTOR^Ser2448^ expression induced by overexpression of BDH2 (Fig. 7b). Furthermore, the 740Y-P pretreatment downregulated the expression levels of cleaved caspase-3, cleaved PARP, and LC3-II and upregulated the expression level of p62 in GC cells expressing BDH2 (Fig. 7c). Consistent with these results, BDH2-induced apoptosis and autophagy were decreased after 740Y-P pretreatment (Fig. 7d-i). Accordingly, IHC revealed that BDH2 overexpression downregulated the expression of p-Akt^Ser473^ and p-mTOR^Ser2448^ in xenografted tumours (Fig. 7j). These results showed that BDH2 enhanced apoptosis and autophagy of GC cells via blocking PI3K/Akt/mTOR signalling.

Next, we elucidated the effect of ROS accumulation on the PI3K/Akt/mTOR pathway. As shown in Fig. S4B, pretreatment with NAC reversed the change in expression levels of p-Akt^Ser473^ and p-mTOR^Ser2448^ in BDH2-overexpressing cells. In summary, these data indicated that BDH2 inhibits the activity of the PI3K/Akt/mTOR pathway by inducing accumulation of ROS in GC cells.

## Discussion

In this study, we found that BDH2 was significantly down-regulated in GC tissues and that BDH2 expression correlated with adverse clinicopathological parameters in GC. Furthermore, Kaplan–Meier analysis showed that GC patients with low expression of BDH2 had a poor prognosis. Hypermethylation of the gene promoter region is one of the important mechanisms of gene inactivation. We used MethPrimer (http://www.urogene.org/methprimer/) and did not find any CpG island in the BDH2 promoter region, indicating that the down-regulation or deletion of the BDH2 gene in GC tissues may not be related to DNA promoter methylation. The occurrence of GC is the result of a combination of multiple factors. For example, long-term chronic stimulation of various inflammatory factors is a key factor leading to the occurrence of GC [23, 24]. Previous studies have shown that sustained inflammation can cause down-regulation of BDH2 expression [25]. Therefore, we speculate that long-term continuous stimulation of inflammatory factors during the development of GC is an important cause of down-regulation of BDH2 expression in GC tissues.

In the present study, we identified that overexpression of BDH2 inhibited GC cell growth in vitro and in vivo. Iron participates in various biological functions, such as cell proliferation, growth, and ferroptosis [26]. Evidence from recent studies showed that the mammalian siderophore is an important regulator of cellular iron homeostasis [27]. Notably, previous studies have shown that biosynthesis of the mammalian siderophore (2,5-DHBA) is catalyzed by BDH2 [27]. However, whether the biological function induced by BDH2 is related to intracellular iron levels remains unclear. Here, we used a colorimetric assay to measure cytoplasmic iron concentration in GC cells. Our results indicated that overexpression of BDH2 had little effect on iron levels in GC cells (Fig. S5).

AMPK, MAPK/Erk1/2, and PI3K/Akt/mTOR signalling pathways are classic apoptosis and autophagy pathways [28–30]. Previous studies have shown that BDH2 inhibits HCC cell growth, proliferation, and migration by inducing apoptosis and autophagy [31]. However, the specific molecular mechanisms of BDH2-induced apoptosis and autophagy remained unclear. Thus, we explored whether BDH2 participates in the regulation of three classic pathways in GC cells. Western blotting indicated that the phosphorylation levels of Akt^Ser473^ and mTOR^Ser2448^ were downregulated in BDH2-overexpressing cells. However, no significant changes in the expression of p-AMPK, p-p38 MAPK, p-JNK, or p-ERK were found in BDH2-overexpressing cells. Additionally, expression of p-Akt^Ser473^ and p-mTOR^Ser2448^ was reversed after pretreatment with 740Y-P. Interestingly, pretreatment with 740Y-P also significantly reversed the formation of autophagic flux and enhancement of LC3-II. Moreover, 740Y-P pretreatment reduced BDH2-induced cell death. These results indicated that BDH2 suppressed the growth of GC cells via inhibition of PI3K/Akt/mTOR signalling.

ROS play a key role in the induction of apoptosis and autophagy [32]. ROS are important signalling molecules in cells, which participate in multiple signalling pathways. A recent report revealed that ROS participate in the induction of caspase-independent cell death in macrophages [33, 34]. Moreover, ROS induce autophagy and apoptosis of tumour cells by inhibiting pathways such as PI3K/Akt and JNK, thereby suppressing the malignant phenotype of tumour cells [35, 36]. In this study, a significant increase in ROS generation was detected by flow cytometry and DCFH-DA in the BDH2-overexpressing group, which was clearly inhibited by a ROS scavenger (NAC). We found that BDH2 overexpression triggered apoptosis and autophagy in GC cells by inducing ROS production. However, treatment with NAC drastically rescued apoptotic cell death. In addition, NAC pretreatment remarkably inhibited autophagic flux and conversion of LC3-I to LC3-II. Furthermore, NAC reversed the expression levels of p-Akt^Ser473^ and p-mTOR^Ser2448^ in BDH2-overexpressing cell lines. Taken together, these results implied that BDH2-induced apoptosis and autophagy were triggered through ROS-mediated PI3K/Akt/mTOR pathways.

ROS produced by oxidative stress are regulated by Nrf2 and its downstream target genes including HO-1 and NQO1 [37–40]. In healthy cells, Nrf2 binds to Keapl in the cytoplasm, which then recruits E3 ubiquitin ligase Cullin 3, thereby facilitating rapid degradation of Nrf2 by proteasomes [41–43]. However, under oxidative stress conditions, Keap1 is uncoupled from Nrf2, allowing Nrf2 to transfer into the nucleus and bind to the ARE, thereby activating downstream antioxidants to increase cellular antioxidant activity. Nrf2 is highly expressed in all kinds of tumour tissues including GC, and cancer cells employ the cytoprotective action of Nrf2 to counter a microenvironment that is not conducive for tumour growth [44–47]. Several mechanisms have been reported for continuous activation of Nrf2 in GC. A nonsynonymous somatic mutation (G333C) was detected in the first repeat of the double glycine repeat/Kelch domain, which does not repress the activity of Nrf2 [48]. The increased expression of Nrf2 helps to maintain ROS levels below a toxic threshold to escape death in cancer cells [49]. Recent studies have shown that inhibition of Nrf2 activity suppresses tumour growth and enhances the therapeutic efficiency of chemotherapeutic drugs against cancer [50–53]. Therefore, under different pathological conditions, analysing Nrf2 sheds light on cancer prevention and treatment. In the present study, we found that BDH2 overexpression inhibited ARE-mediated antioxidant gene expression. Moreover, BDH2 downregulated Nrf2 expression through its protein stability rather than transcription. Co-IP verified the effect of BDH2 on binding of Keap1 and Nrf2. Our results suggested that BDH2 stabilises the binding of Keap1 and Nrf2 to promote ubiquitination/degradation of Nrf2 in GC cells.

## Conclusions

**Fig. 8:**
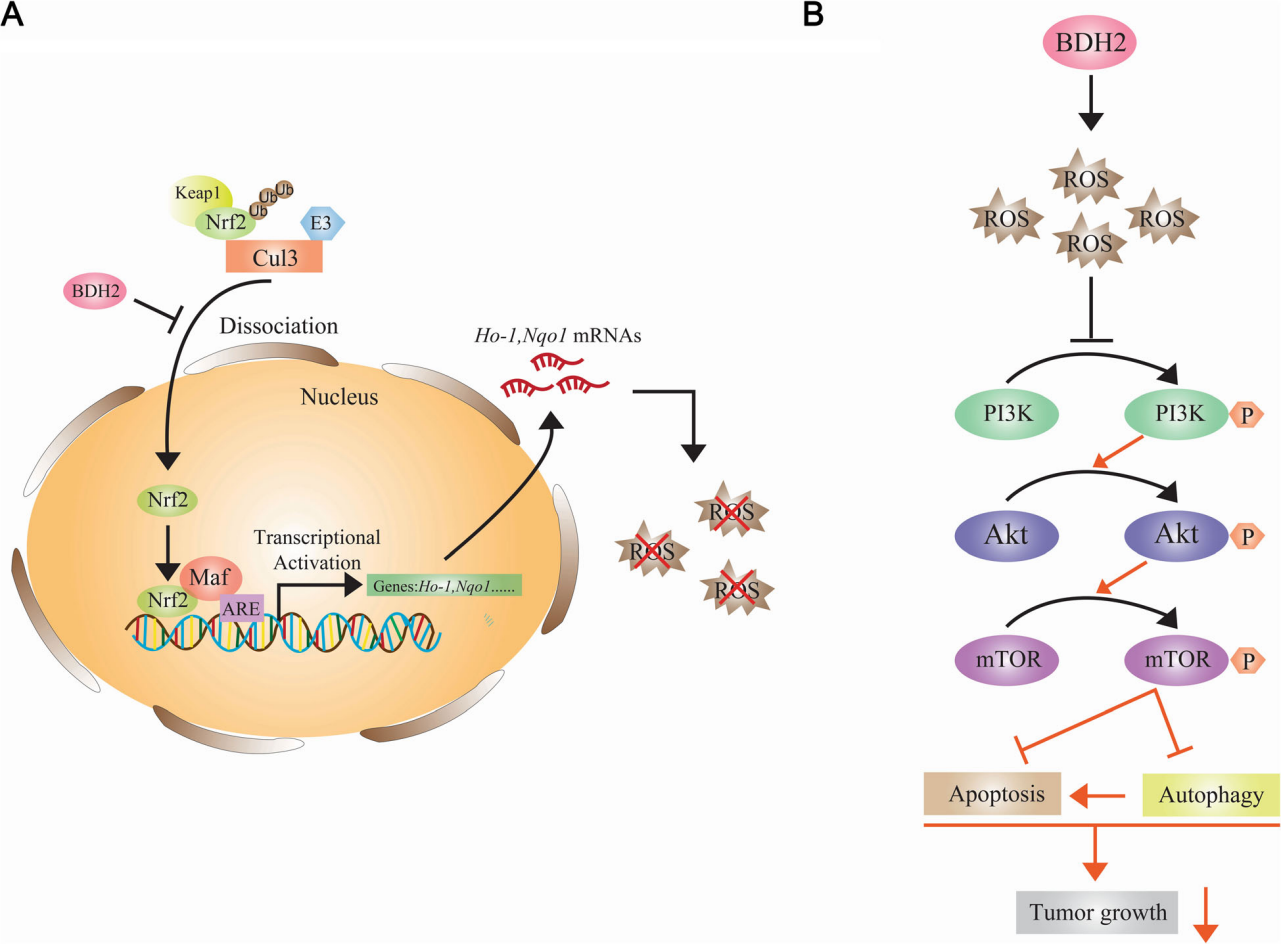
Model illustrating the anti-tumour activity of BDH2. **a** Schematic representation of the mechanism of BDH2 regulation of Nrf2. **b** Schematic representation of the mechanism by which BDH2 overexpression promotes cell death and autophagy in GC

In conclusion, we have identified a novel tumour suppressor gene, BDH2, which is frequently downregulated in GC. BDH2-induced ROS accumulation suppresses phosphorylation of Akt^Ser473^ and mTOR^Ser2448^ by promoting ubiquitination of Nrf2, thereby inhibiting the growth of GC cells and playing a tumour-suppressive role (Fig. 8). These findings indicate that BDH2 may be a potential new target for the treatment of GC.

## Supplementary information

**Additional file 1 Table S1**. Sequences of primers used for amplification of target genes. **Fig. S1** Inhibition of autophagy inhibits BDH2-induced apoptosis. A In BDH2-overexpressing SGC7901 and BGC823 cells, the expression levels of LC3-II, p62, cleaved caspase-3, and PARP were determined by western blotting after treatment with the autophagy inhibitor 3-MA. B In BDH2-overexpressing SGC7901 and BGC823 cells, cell viability was measured by CCK8 assays in the absence or presence of 3-MA. C In BDH2-overexpressing SGC7901 and BGC823 cells, apoptosis was assessed by flow cytometry in the absence or presence of 3-MA. *p < 0.05, **p < 0.01. **Fig. S2** BDH2 overexpression triggers ROS generation. Left panel: Detection of intracellular ROS levels by fluorescence microscopy (magnification, × 200, scale bars, 20 μm). Right panel: Quantitative representation of ROS production indicated by fluorescence signal intensities. *p < 0.05; ** *p* < 0.01. **Fig. S3** Effect of BDH2 on Keap1 and Nrf2 mRNA levels. The mRNA levels of Keap1 and Nrf2 were measured by qRT-PCR. Results are presented as means ± S.D. (*n* = 3); ns, not significant. **Fig. S4** BDH2-induced ROS have an important role in the PI3K/Akt/mTOR pathway. A Levels of relevant signalling pathway proteins in BDH2-overexpressing SGC7901 and BGC823 cells were examined by western blotting. B Protein expression levels of p-Akt^Ser473^ and p-mTOR^Ser2448^ were detected in the presence or absence of NAC by western blotting. **Fig. S5** Effect of BDH2 on intracellular iron levels. Cells expressing BDH2 or vector were analyzed for intracellular iron concentration by colorimetry. Results are presented as means ± S.D. (*n* = 3); ns, not significant.

## Data Availability

The datasets used or analysed during the current study are available from the corresponding author on reasonable request.

## Abbreviations

GC: Gastric cancer
BDH2: 3-Hydroxy butyrate dehydrogenase 2
Baf-A1: Bafilomycin A1
3-MA: 3-methyladenine
ARE: Antioxidant response elements
Nrf2: Nuclear factor erythroid 2-related factor 2
HO-1: Heme oxygenase 1
NQO1: NAD(P)H–quinone oxidoreductase 1
ROS: Reactive oxygen species
Keap1: Kelch-like ECH-associated protein 1
NAC: N-acetylcysteine
