# Supplementary material for: BDH2 triggers ROS-induced cell death and autophagy by promoting Nrf2 ubiquitination in gastric cancer
Source: J Exp Clin Cancer Res. 2020 Jun 30;39:123. doi: 10.1186/s13046-020-01620-z (PMC7325376; doi:10.1186/s13046-020-01620-z)
Supplement: Supplementary file 1 — Additional file 1 Table S1. Sequences of primers used for amplification of target genes. Fig. S1 Inhibition of autophagy inhibits BDH2-induced apoptosis. A In BDH2-overexpressing SGC7901 and BGC823 cells, the expression levels of LC3-II, p62, cleaved caspase-3, and PARP were determined by western blotting after treatment with the autophagy inhibitor 3-MA. B In BDH2-overexpressing SGC7901 and BGC823 cells, cell viability was measured by CCK8 assays in the absence or presence of 3-MA. C In BDH2-overexpressing SGC7901 and BGC823 cells, apoptosis was assessed by flow cytometry in the absence or presence of 3-MA. *p < 0.05, **p < 0.01. Fig. S2 BDH2 overexpression triggers ROS generation. Left panel: Detection of intracellular ROS levels by fluorescence microscopy (magnification, × 200, scale bars, 20 μm). Right panel: Quantitative representation of ROS production indicated by fluorescence signal intensities. *p < 0.05; ** p < 0.01. Fig. S3 Effect of BDH2 on Keap1 and Nrf2 mRNA levels. The mRNA levels of Keap1 and Nrf2 were measured by qRT-PCR. Results are presented as means ± S.D. (n = 3); ns, not significant. Fig. S4 BDH2-induced ROS have an important role in the PI3K/Akt/mTOR pathway. A Levels of relevant signalling pathway proteins in BDH2-overexpressing SGC7901 and BGC823 cells were examined by western blotting. B Protein expression levels of p-AktSer473 and p-mTORSer2448 were detected in the presence or absence of NAC by western blotting. Fig. S5 Effect of BDH2 on intracellular iron levels. Cells expressing BDH2 or vector were analyzed for intracellular iron concentration by colorimetry. Results are presented as means ± S.D. (n = 3); ns, not significant. [file 13046_2020_1620_MOESM1_ESM.docx]

BDH2 triggers ROS-induced cell death and autophagy by promoting Nrf2 ubiquitination in gastric cancer

Jia-Zhou Liu^1,2^, Yi-Lin Hu^1,2^, Ying Feng^1,2^, Yun Jiang^2^, Yi-Bing Guo^2^, Yi-Fei Liu^3^, Xi Chen^1,2^, Jun-Ling Yang^2^, Yu-yan Chen^1,2^, Qin-Sheng Mao^1^, and Wan-Jiang Xue^1, 2^

**Supplementary information**

**Supplementary Table S1. Sequences of primers used for amplification of target genes**

| Gene primer nucleotide sequence |
| --- |
| BDH2 Forward: 5′-GCTTCCAGCGTCAAAGGAGTT-3′  Reverse: 5′-CAGTTGCGAATCTTCCCGTC-3′  NQO1 Forward: 5′-TGCAGCGGCTTTGAAGAAGAAAG-3′  Reverse: 5′-TCGGCAGGATACTGAAAGTTCGC-3′  HO-1 Forward: 5′-TCTCTTGGCTGGCTTCCTTAC-3′  Reverse: 5′-GGCTTTTGGAGGTTTGAGACA-3′  Keap1 Forward: 5′-TGGCCAAGCAAGAGGAGTTC-3′  Reverse: 5′-GGCTGATGAGGGTCACCAGTT-3′  Nrf2 Forward: 5′-AACCAGTGGATCTGCCAACTACT C-3′  Reverse: 5′-CTGCGCCAAAAGCTGCAT-3′  β-actin Forward: 5′-CATGGAGTCCTGTGGCATC-3′  Reverse: 5′-GGAGCAATGATCTTGATCTTC-3′ |

**Supplementary Figure Legends**


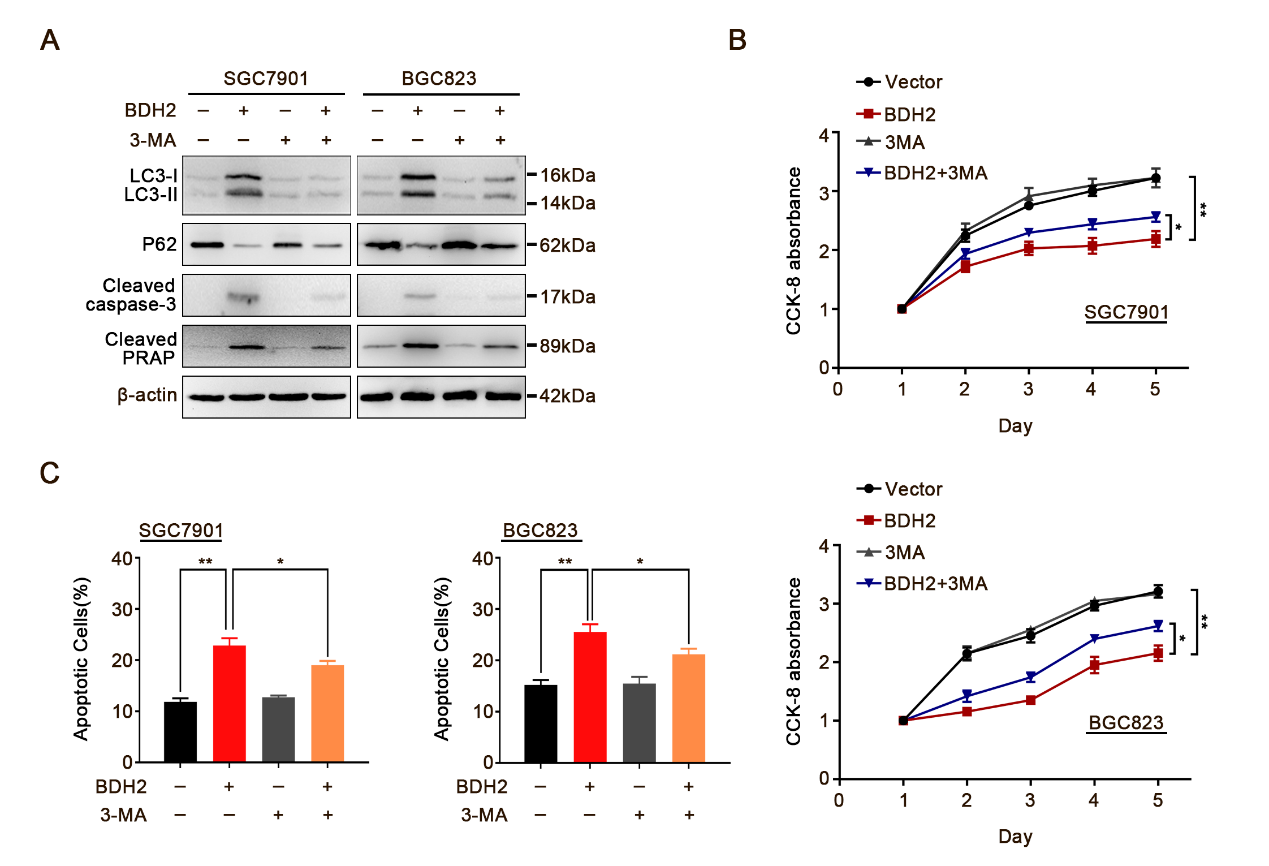


**Fig. S1 Inhibition of autophagy inhibits BDH2-induced apoptosis.** **A** In BDH2-overexpressing SGC7901 and BGC823 cells, the expression levels of LC3-II, p62, cleaved caspase-3, and PARP were determined by western blotting after treatment with the autophagy inhibitor 3-MA. **B** In BDH2-overexpressing SGC7901 and BGC823 cells, cell viability was measured by CCK8 assays in the absence or presence of 3-MA. **C** In BDH2-overexpressing SGC7901 and BGC823 cells, apoptosis was assessed by flow cytometry in the absence or presence of 3-MA. **p* < 0.05, ***p* < 0.01.


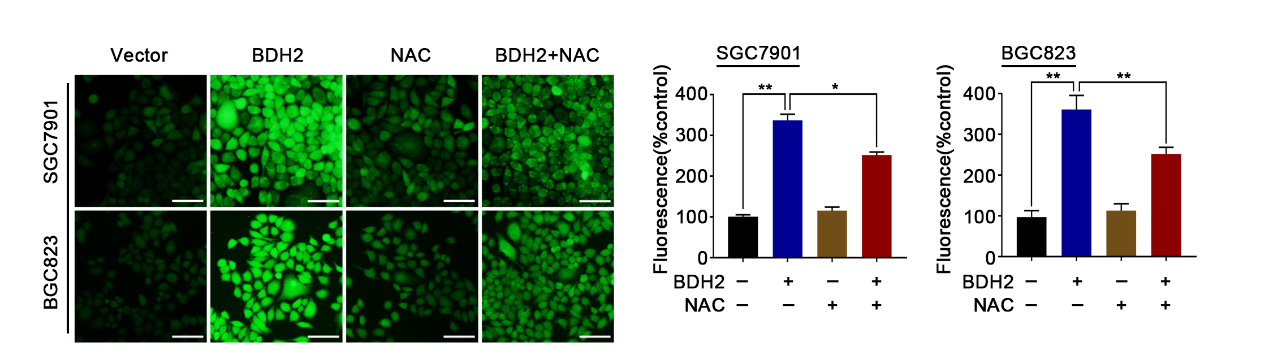


**Fig. S2 BDH2 overexpression triggers ROS generation.** Left panel: Detection of intracellular ROS levels by fluorescence microscopy (magnification, ×200, scale bars, 20 μm). Right panel: Quantitative representation of ROS production indicated by fluorescence signal intensities. **p* < 0.05; ** *p* < 0.01.


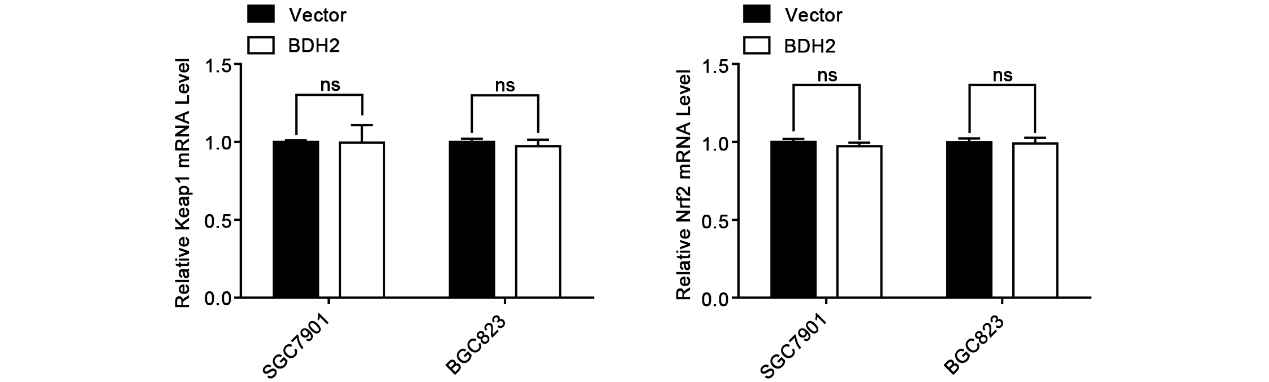


**Fig. S3 Effect of BDH2 on Keap1 and Nrf2 mRNA levels.** The mRNA levels of Keap1 and Nrf2 were measured by qRT-PCR. Results are presented as means ± S.D. (n = 3); ns, not significant.


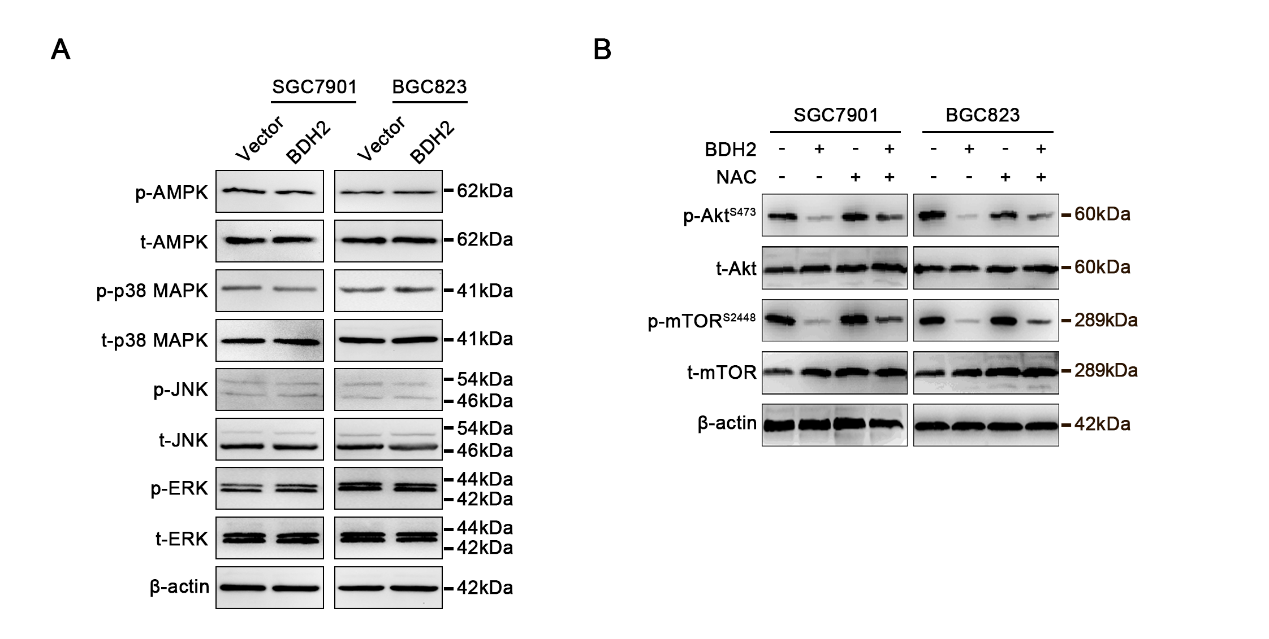


**Fig. S4 BDH2-induced ROS have an important role in the PI3K/Akt/mTOR pathway.** **A** Levels of relevant signalling pathway proteins in BDH2-overexpressing SGC7901 and BGC823 cells were examined by western blotting. **B** Protein expression levels of p-Akt^Ser473^ and p-mTOR^Ser2448^ were detected in the presence or absence of NAC by western blotting.


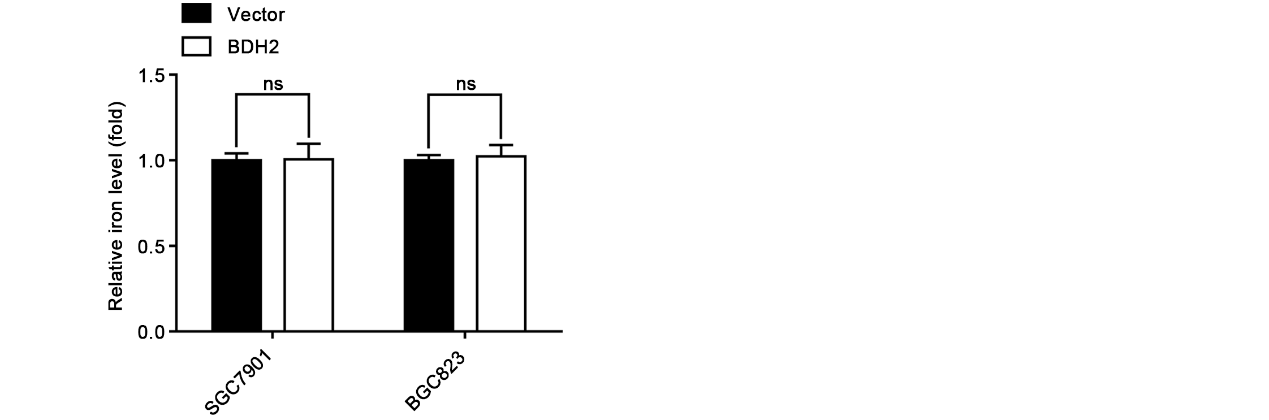


**Fig. S5 Effect of BDH2 on intracellular iron levels.** Cells expressing BDH2 or vector were analyzed for intracellular iron concentration by colorimetry. Results are presented as means ± S.D. (n = 3); ns, not significant.
